# Measuring Indoor Occupancy through Environmental Sensors: A Systematic Review on Sensor Deployment

**DOI:** 10.3390/s22103770

**Published:** 2022-05-16

**Authors:** Alma Rosa Mena, Hector G. Ceballos, Joanna Alvarado-Uribe

**Affiliations:** School of Engineering and Sciences, Tecnologico de Monterrey, Monterrey 64849, Mexico; a00834070@tec.mx (A.R.M.); joanna.alvarado@tec.mx (J.A.-U.)

**Keywords:** indoor occupancy, environmental sensors, machine learning, deployment

## Abstract

The COVID-19 pandemic has changed our common habits and lifestyle. Occupancy information is valued more now due to the restrictions put in place to reduce the spread of the virus. Over the years, several authors have developed methods and algorithms to detect/estimate occupancy in enclosed spaces. Similarly, different types of sensors have been installed in the places to allow this measurement. However, new researchers and practitioners often find it difficult to estimate the number of sensors to collect the data, the time needed to sense, and technical information related to sensor deployment. Therefore, this systematic review provides an overview of the type of environmental sensors used to detect/estimate occupancy, the places that have been selected to carry out experiments, details about the placement of the sensors, characteristics of datasets, and models/algorithms developed. Furthermore, with the information extracted from three selected studies, a technique to calculate the number of environmental sensors to be deployed is proposed.

## 1. Introduction

Occupancy information refers to the presence of occupants in a building, their movement, and their behavior. The occupancy information can be used to optimize a building’s energy consumption and reduce energy waste [1]. Furthermore, in a world where social distancing and space occupancy limitation policies have been enforced due to the COVID-19 pandemic, monitoring systems become tools not only to improve the management of spaces but also to save human lives [2].

To acquire occupancy-related information, there are different sensing approaches. For instance, intrusive sensors, such as cameras and pattern recognition, are used to count people; nevertheless, personal privacy is one problem during implementation. In contrast, the non-intrusive sensors types, such as passive infrared (PIR), ultrasonic, and acoustic sensors, can only be used to determine whether the room is occupied rather than determining the actual number of occupants [3].

**Figure 1:**
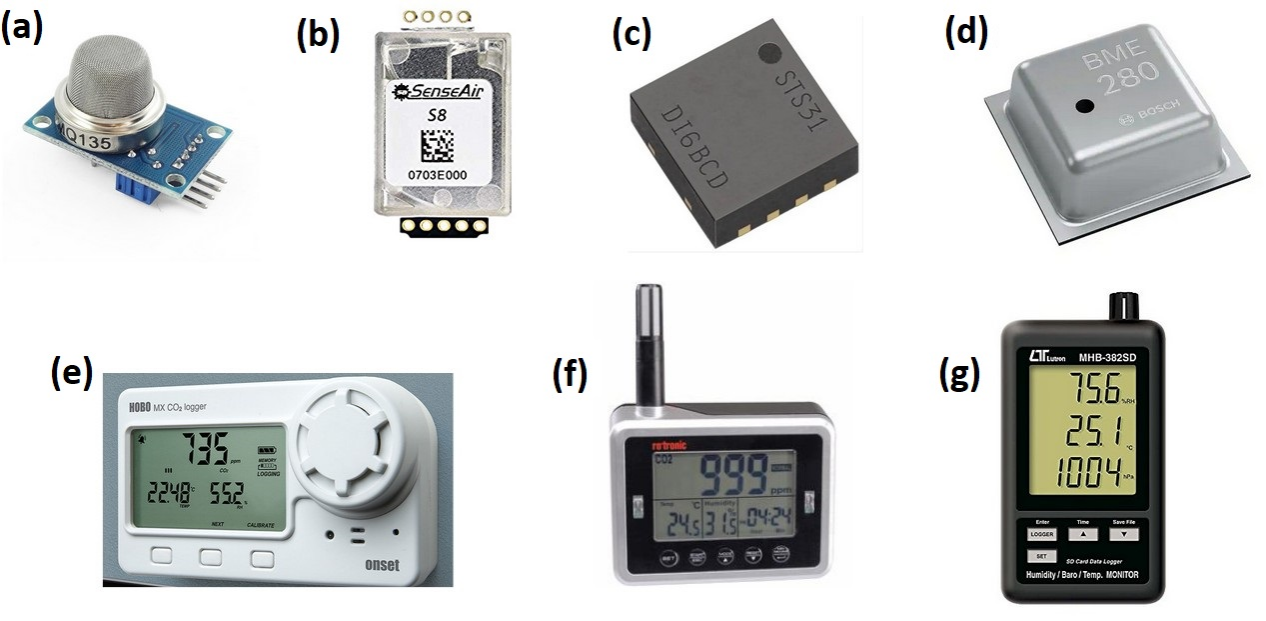
Environmental sensors used for collecting occupancy-related information. (**a**) CO${}_{2}$ sensor MQ135. (**b**) CO${}_{2}$ sensor SenseAir S8. (**c**) Temperature sensor SENSIRION STS31. (**d**) Temperature, RH, Pressure sensor BME280. (**e**) CO${}_{2}$ sensor HOBO MX1102. (**f**) CO${}_{2}$, temperature and RH sensor CL11. (**g**) Barometric pressure sensor MHB-382SD.

Environmental sensors are frequently used in occupancy modeling because of their non-intrusive nature, their flexibility in sensor selection and combination, and their ability to provide continuous data streams for real-time occupancy modeling [4]. Most environmental sensors can measure CO${}_{2}$ concentration, temperature, relative humidity (RH), airspeed, particulate matter (PM), and volatile organic compounds (VOC) [5]. Figure 1 presents the commonly used sensors for CO${}_{2}$, temperature, RH, and barometric pressure. The available CO${}_{2}$ sensors are MQ135 [6], CL11 (also measure temperature and RH) [7,8,9], SenseAir S8 [10], and HOBO MX1102 Zhou2020, among others. To measure temperature, RH, and barometric pressure, some commercial sensors are the SENSIRION STS31 [11], BME280 [2,12], and MHB-382SD [8].

Occupancy modeling approaches are divided into categories based on their level of accuracy. These approaches include binary detection of the occupant’s presence (occupancy detection) and counting the number of occupants (occupancy estimation) [4]. However, some authors have also developed models to estimate the levels of occupation as well as the social interaction [13] and status (to determine whether a person is alive or not) that a human being has [14].

Regarding practical implementations of occupancy detection and prediction, researchers have proposed various models that involve common statics models. Some models used are the Hidden Markov Model (HMM) and its variations [15,16,17], models based on Bayes’ theorem [7,18], supervised Machine Learning models, such as Support Vector Machine (SVM) [19,20], Random Forest (RF) [21,22], and the popular Artificial Neural Networks (ANN) as well as their variants [23,24]. Furthermore, some researchers have also proposed combining multiple environmental parameters in the models to obtain higher precision and accuracy [25,26,27].

Existing reviews articles have performed a comprehensive overview of current solutions for occupancy estimation and detection using different categories of sensors [28,29,30,31]. All of them discuss and compare the characteristics of the sensors, both the advantages and the disadvantages. Other reviews address the modeling techniques and evaluation for occupancy inference [32,33,34]. Furthermore, some authors have carried out an extensive review incorporating the type of sensors, prediction models, and potential uses of low-cost sensors in buildings [35,36,37]. Nevertheless, none of them address the installation aspects and the number of sensors needed to place in a specific enclosed space. As a consequence, an intuitive deployment caused an increase in time and cost.

Hence, the purpose of this systematic review is to identify articles that present indoor occupancy approaches using at least one environmental sensor. That is, the aim is to find out indoor occupancy models, the number of sensors installed in occupancy environments, a description of the enclosed spaces, and the details about sensor deployment. The main interest of carrying out this research lies in reviewing how sensors are installed in test-bed scenarios and the least number of sensors required to generate adequate data for analysis. This information can be beneficial for future research works that are focused on indoor occupancy.

The content of this work is organized as follows: Section 2 presents the methodology used to perform this systematic review. Section 3 provides the results obtained from this review, and Section 4 gives the discussion of the results. Finally, Section 5 presents the conclusions of this study.

## 2. Materials and Methods

This systematic review was conducted in accordance with the PRISMA (Preferred Reporting Items for Systematic Review and Meta-Analisis) checklist. The PRISMA statement was designed to help systematic reviewers transparently report why the review was done, what the authors did, and what they found. The PRISMA statement comprises a checklist of 27 items recommended for reporting in systematic reviews and an “explanation and elaboration” paper that provides additional reporting guidance for each item along with sample reports [38].

Furthermore, a second methodology developed by Kofod-Petersen [39] was considered in this study. The Kofod-Petersen method helps researchers conduct a structured literature review within Computer Science.

The review process has been broken down into several steps. First of all, relevant questions have been identified, and a specific strategy has been followed to answer them. This strategy is described along with the specific search strings and keywords used. Next, the inclusion and exclusion criteria for the selection of relevant literature are defined. Subsequently, data extraction and synthesis are carried out based on the already conducted search. Finally, the risk of bias and limitations of this systematic review process are discussed. The aforementioned steps for this systematic review are described in detail in the following subsections.

### 2.1. Research Questions

The purpose of this systematic review is to establish a relationship between the number of environmental sensors to collect data and the enclosed space in which occupancy levels will be estimated. The objectives for establishing this relationship are:

- Identify the environmental variables used.
- Obtain the number of sensors installed.
- Obtain the details of the sensor deployment.
- Obtain the algorithms and models used to calculate, predict, or estimate occupancy.

For this systematic review, some questions were identified:

- RQ1: How many sensors per square meter are necessary to install within a room in order to estimate occupancy levels in real time using only environmental sensors (air temperature, barometric pressure, relative humidity)?
- RQ2: Is it essential to add another environmental variable or non-intrusive sensor to improve the classification?
- RQ3: How should the sensors be distributed within the indoor place?
- RQ4: Can data fusion improve the performance of predictors?
- RQ5: Is it possible to use unlabeled data to estimate occupancy levels?

### 2.2. Search Process

For this systematic review, the well-known scientific database SCOPUS [40] is used to find relevant literature. The search process was initiated on 5 July 2021 and concluded on 10 September 2021. The search results were saved in SCOPUS and the selected publications were downloaded and imported to JabRef reference manager.

The main search keywords used were “*Occupancy estimation*”, “*Occupancy detection*”, “*Occupancy Levels*”, “*Occupancy*” as well as “*environmental variables*” and “*environmental sensors*”.

### 2.3. Inclusion and Exclusion Criteria

The inclusion and exclusion criteria used for screening and selecting relevant literature from the search results are defined in Table 1.

### 2.4. Study Selection

**Figure 2:**
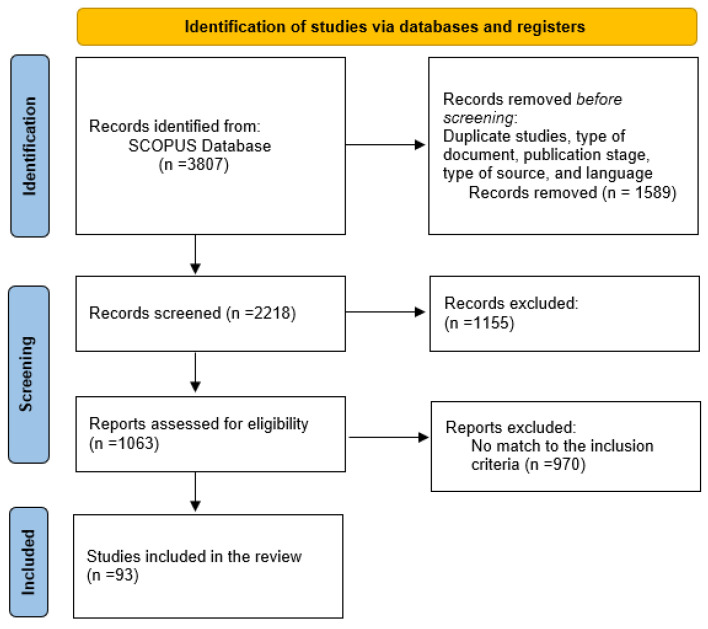
PRISMA flow diagram for study selection.

For the selection of articles, the criteria involved the revision of the document title, the abstract, and the skimming of the article. Furthermore, the inclusion and exclusion criteria were also applied. A selection process based on the PRISMA flowchart [38], presented in Figure 2, was also used.

The selected keywords provided around 3807 publications. Among these articles, 1063 mainly focus on occupancy prediction, estimation, and detection. Finally, 93 studies were selected as only these studies fulfilled the search and inclusion criteria (Table 2).

### 2.5. Data Extraction and Synthesis

The general information for the registration of articles should include information regarding the type of sensor and quantity, dimension of the enclosed space, design use of the room, the data collection period, and the model or method used to estimate occupancy levels as well as the accuracy obtained.

The data extracted from selected literature are tabulated in an Excel spreadsheet, according to the following structure:

- Title and abstract of the literature;
- Author(s);
- Database type (available or private);
- Publication year;
- Type of sensor;
- Quantity of sensors;
- Type of place;
- Place dimensions;
- Description of the sensor deployment process;
- Test time;
- University–Country;
- Machine Learning algorithms;
- Data fusion methods;
- Results.

After the data extraction step, the extracted data are analyzed to answer the research questions. For RQ1 and RQ2, the types of sensors used to estimate/detect indoor occupancy are listed, and the respective number of sensors installed in that enclosed space are analyzed. For RQ3, an analysis of the sensor deployment is reported, whereas for RQ4, the studies that utilized data fusion methods are listed. Finally, for RQ5, Machine Learning methods are analyzed based on their trend over time as well as the characteristics of the used dataset.

### 2.6. Risk of Bias

The risk of bias started during the initial query search in the database as the search produced only the literature that was published between 2009 and 2021. Moreover, the possible subjectivity of the inclusion and exclusion criteria defined by the authors can also increase the bias in the selection process. Furthermore, there could be publications that have been missed during the search process as the search was only performed through the SCOPUS database.

In addition to the aforementioned biases, this systematic review has focused on publications involving at least one environmental sensor to estimate/detect indoor occupancy.

## 3. Results

This section presents the findings from extracted data based on the questions provided in Section 2. In the “Study Characteristics” section, a brief description of the selected publications is presented. Next, RQ1 and RQ2 are answered in the respective sections “Type of Sensors and Indoor Place Characteristics” and “Place Dimension vs. Total of Sensors Deployed.” This is followed by the “Sensor Deployment Specifications” section, where the proposed locations and the height of the sensors are detailed (RQ3). The “Data Fusion” section addresses the methods proposed by different authors and answers RQ4. Finally, the “Description of Algorithms and Datasets” section discusses the approaches for occupancy estimation/detection, answering RQ5.

### 3.1. Study Characteristics

**Figure 3:**
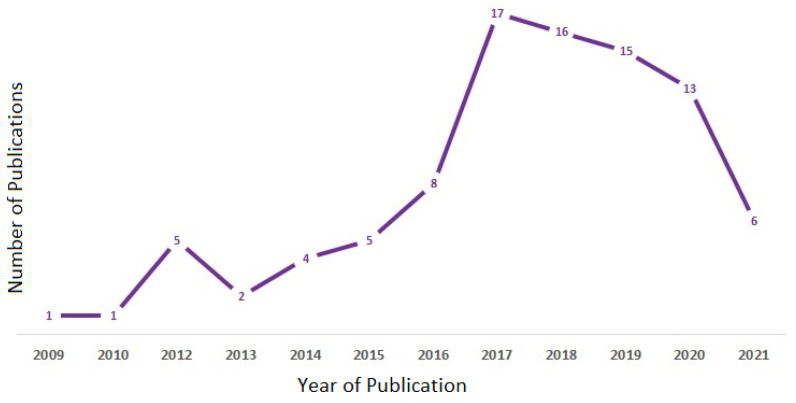
Global annual publications on indoor occupancy estimation/detection using environmental variables.

Interest in indoor occupancy detection and estimation using environmental sensors has been growing over the years and has led to an increase in the annual output of articles in the related domain from 2009 to 2021 (Figure 3). Lam et al. [41], pioneer in this domain, developed algorithms to calculate the number of occupants based on the analysis of the environmental data obtained. Later, in the year 2012, the research interest in this domain had a strong increase. In subsequent years from 2012 to 2017, the number of scientific publications on occupancy estimation increased significantly. The year with the most publications is 2017 (17 papers), while in the following years, the publication trend has slowly decreased.

**Figure 4:**
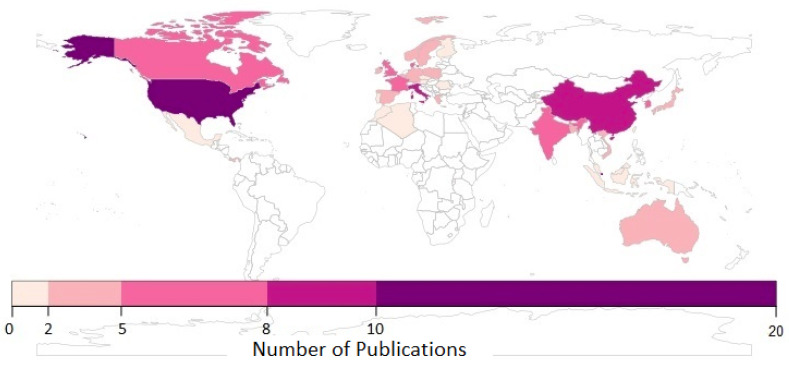
Publications on indoor occupancy by country.

Figure 4 shows the distribution of publications on indoor occupancy by country. In terms of publications by country, the United States of America (USA) has published the most studies (20), representing 21.5% of the total publications in this field of research. The USA is followed by Singapore and China with respective publications of 10 (10.75%) and 9 (9.67%). The average citations within SCOPUS of articles from USA, Singapore, and China are 48.2, 31.3, and 29, respectively. Notably, the USA holds the leading position in the research area of indoor occupancy estimation/detection using environmental variables.

**Figure 5:**
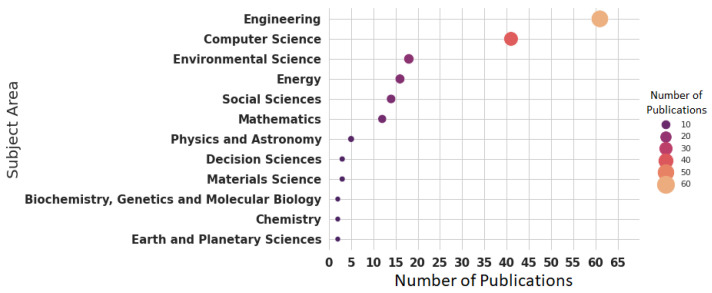
Publications on indoor occupancy by subject area.

Regarding the subject areas in which the studies are published (Figure 5), most of the studies are concentrated in the field of Engineering (65.59%) and Computer Science (44.08%), followed by Environmental Science (19.35%) and Energy (17.20%). However, the publications related to subjects such as Earth and Planetary Science, Chemistry, Biochemistry-Genetics, and Molecular Biology are scarce and represent only 2.15% of the total number of publications.

**Figure 6:**
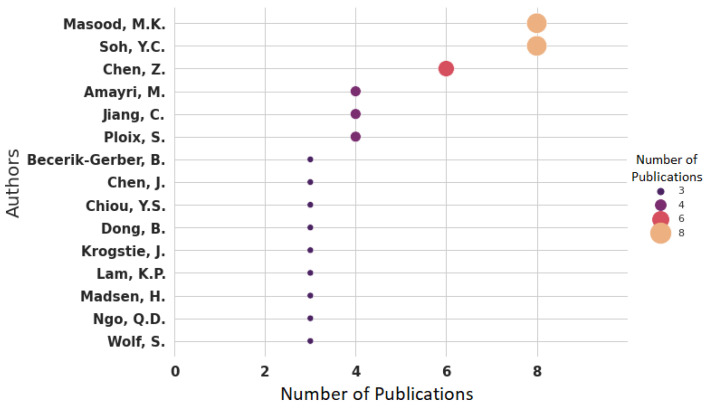
Publications on indoor occupancy estimation by author.

Moreover, there are about 160 authors involved in this research area. Of these authors, the number of discovered authors who have published at least three articles and are included in this systematic review is 15 (9.35%). In total, 100 authors (62.5%) have only one publication, while 42 authors (26.25%) have two publications, indicating that a limited group of researchers (three publications, representing 1.87%) have focused on this domain. The top 15 authors who have published at least three papers in the domain of indoor occupancy estimation are shown in Figure 6. The top two researchers, M.K. Massod and Y.C. Soh, are collaborating closely.

**Figure 7:**
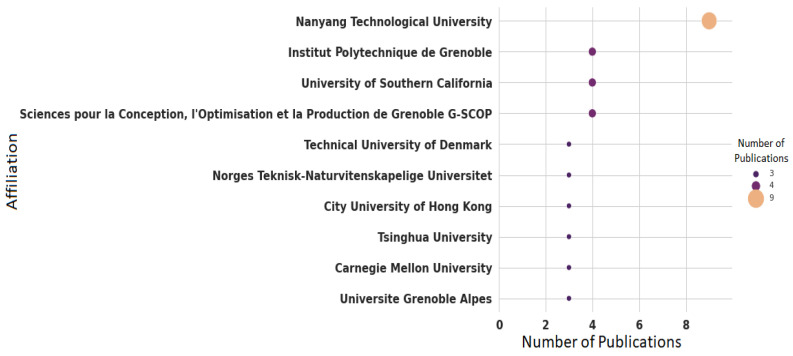
Publications on indoor occupancy estimation by affilation.

The institution with the highest number of publications related to indoor occupancy estimation is Nanyang Technological University (nine publications, representing 9.67%) in Singapore, followed by Institut Polytechnique de Grenoble (four publications, representing 4.30%) in France, University of Southern California (four publications, representing 4.30%) in USA, and Sciences pour la Conception, l’Optimisation et la Production de Grenoble G-SCOP (four publications, representing 4.30%) in France. Nevertheless, there are more than 130 institutions that have conducted research in this field, and of those 130, 99 institutions have only one publication, 25 institutions have two, and 6 institutions have three publications. Figure 7 presents the top 10 institutions that have at least three publications.

**Figure 8:**
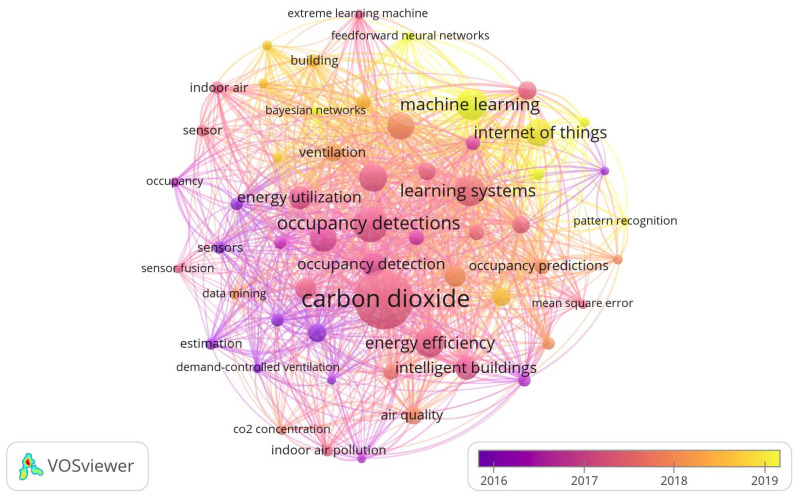
The most relevant keywords of the selected publications.

When reviewing keywords from the literature, the keywords with a minimum co-occurrence equal to five are presented in a network map (Figure 8), which was constructed using the VOSviewer software—version 1.6.17 [42]. The size of the nodes and the words in Figure 8 represent their respective weight. The bigger the node and the word, the greater their weight. The distance between two nodes reflects their strength. That is, a shorter distance reveals a stronger relationship. The line joining two keywords represents that they have appeared together. The thicker the line, the more co-occurrence they have. The nodes with the same color belong to a cluster [43].

The keyword “carbon dioxide” has the highest frequency of 43. Other keywords with a high frequency include “occupancy detection” (24), “learning system” (21), “machine learning” (21), and “energy efficiency” (20). On the other hand, keywords such as “wireless sensor network”, “social interaction”, “information theory”, and “environmental sensor networks” have the lowest frequency of one.

The network map shows that the keyword “carbon dioxide” has a relationship with the keywords “energy efficiency”, “occupancy detection”, “occupancy detections”, “office buildings”, and “building occupancy”.

**Figure 9:**
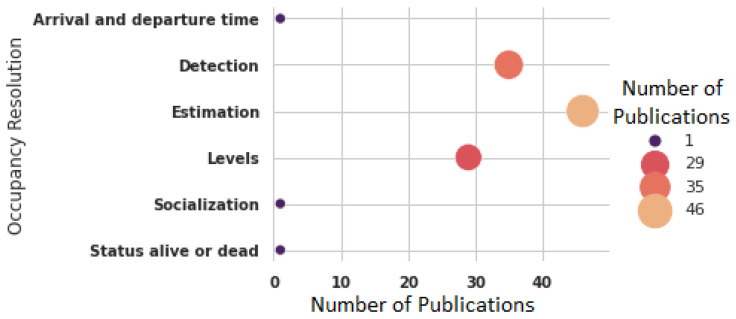
Publications by indoor occupancy resolution.

Finally, Figure 9 shows the research trends of indoor occupancy resolution presenting the changes and evolution of the desired precision over time. The number of occupants estimated in the place is more common; 46 publications (49.46%) were focused on this resolution. The second resolution most common is the detection (binary) of a person, which has been addressed in 35 publications (37.63%), followed by indoor occupancy levels, having been studied in 29 publications (31.18%). It is important to point out that of the 93 publications, 18 focused on more than one resolution.

### 3.2. Type of Sensor and Indoor Place Characteristics

**Figure 10:**
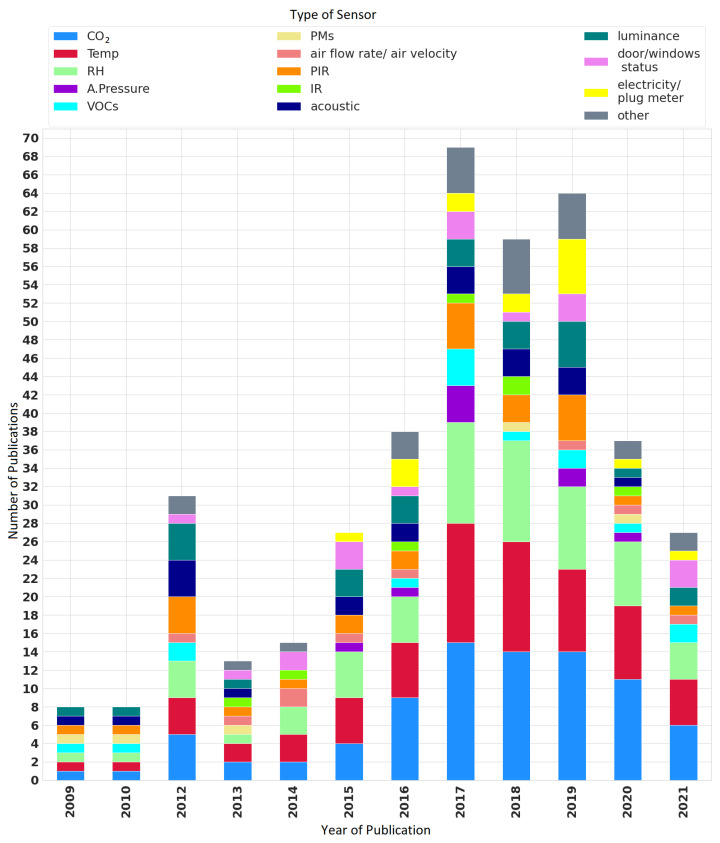
Types of sensors reported in the publications by year.

Indoor occupancy is one of the important sources of information for designing smart buildings. However, challenges such as user privacy, communication limit, and a sensor’s computational capability make it difficult to develop occupancy monitoring systems [44]. Figure 10 shows the types of sensors that have been put into use by year, from 2009 to 2021. The type of sensor that has been used most often over the years is the CO${}_{2}$ sensor. Similarly, sensors that can measure temperature and RH are also widely used for enclosed spaces. In 2017, 15 publications (16.12%) used such sensors to collect environmental data. As secondary sensors, the PIR and light/luminescence sensors have been discussed in 27 publications (29.03%) over the years, followed by the acoustic sensor being discussed in 21 publications (representing 22.5%). While the interest in the PIR sensor increased in the years 2017 and 2019 (five publications, representing 5.37%), in 2019, the interest in the light/luminescence sensor also increased (five publications, representing 5.37%). Previously, the acoustic sensor was the most discussed in 2012 by four publications (4.30%).

Regarding the number of publications reporting on a type of sensor, 84 publications (90.32%) documented the use of the CO${}_{2}$ sensor with other sensors, while 13 publications (13.97%) described the use of the CO${}_{2}$ sensor only. For instance, Zuraimi et al. [45] installed four CO${}_{2}$ sensors within a lecture theater (876 m${}^{2}$), obtaining a root-mean-square error (RMSE) between 19.6% and 27.4%. Other authors who had only installed one CO${}_{2}$ sensor in places with areas between 12 and 40 m${}^{2}$ obtained results with an accuracy value between 69.96% and 99.52% [46,47], between 88% and 94% [10], 86% [48], and an RMSE value of 77% [49]. In places with area between 89 and 186 m${}^{2}$, the results had an accuracy value between 75.5% and 96.5% [50], 85.57% [7], 94% [51], and an RMSE value of 60.44% [52].

On the other hand, 32 publications (34.40%) have only used environmental sensors. Of these 32 publications, only four plublications (4.30%) have used temperature and RH sensors as the main sensors. Their results have accuracies between 83.33% and 87.03% [53], and between 95.2% and 97% [12]. Viani et al. [20,54] installed between 23 and 28 sensors in a place with an area of 1196 m${}^{2}$ to estimate occupancy levels. Their results show that the detection phase was able to correctly recognize more than 82% of the environmental events related to occupancy variations. In addition, Fiebig et al. [55] installed six VOC sensors to detect presence and estimate the occupancy levels in a place with an area of 60 m${}^{2}$, obtaining F1 scores for a binary classifier between 62% and 94%, while for multiclass, scores were between 15% and 94%. On the other hand, Weekly et al. [56] had used eight PM sensors combined with eight airflow sensors in a corridor to detect presence.

**Figure 11:**
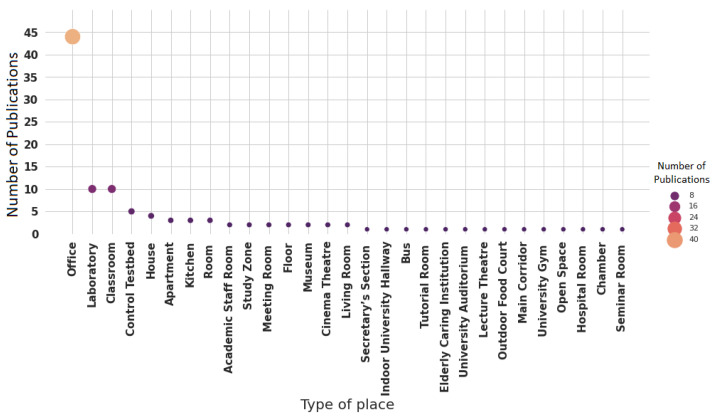
Test-bed scenarios reported in the publications.

Regarding test-bed scenarios (Figure 11), the studies tend to be carried out in offices (44 publications, 47.31%) in which their area varied from 5.03 to 62.92 m${}^{2}$, and 97 to 634.17 m${}^{2}$. The second scenarios are classrooms (10 publications, 10.75%) with areas between 41 and 524.25 m${}^{2}$, and laboratories (10 publications, 10.75%) with a capacity for about four to 70 occupants.

There are particular places that were considered in at least one study (1.07%), such as hospital rooms [57], a bus [58], an elderly caring institution [59], and a university gym [12]. Furthermore, in six studies (6.45%), specially designed places were used to carry out experiments [24,55,60,61,62,63].

### 3.3. Place Dimension vs. Total Number of Sensors Deployed

In various scenarios, the size of the enclosed space varied significantly, causing environmental variables to behave differently. For instance, the physical size of a room is the primary factor in determining its ability to dissipate heat. The larger its area, the lower the temperature rise due to the heat generated in it [64]. Therefore, the number of sensors to be installed in a place should be in accordance with the surface area in order to obtain reliable information.

**Figure 12:**
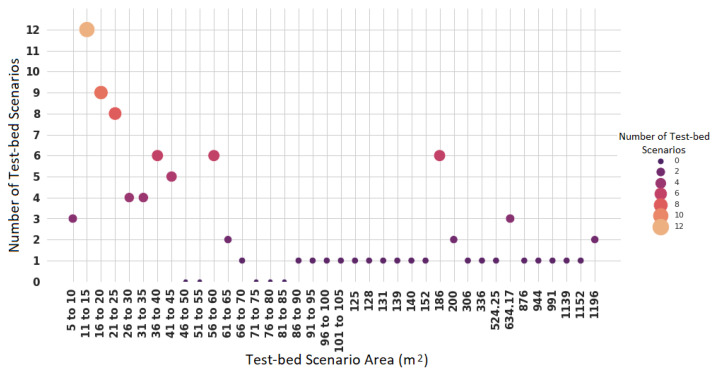
Areas of the test-bed scenarios reported in the literature.

There are around 145 test-bed scenarios in which the authors of the studies considered in this systematic review have conducted experiments. However, not all studies share the dimensions of their enclosure. Figure 12 shows the size of the 65 venues (area in squared meters) and their co-occurrence in research works.

Regarding the size of the test-bed scenarios, the most common scenarios include offices or apartments of 22 m${}^{2}$ or laboratories of 186 m${}^{2}$. The smallest size is of an office of 5 m${}^{2}$, and the largest size is of a museum of 1196 m${}^{2}$. Other mostly documented sizes are offices with an area between 11 and 15 m${}^{2}$ (12 test-bed places, which represent 18.46%). They are followed by spaces with areas between 16 and 20 m${}^{2}$ (nine test-bed places, representing 13.84%) and those with areas between 21 and 25 m${}^{2}$ (eight test-bed places, representing 12.30%). However, there are some studies that have measured and collected data from places with areas between 66 and 152 m${}^{2}$ and larger areas from 306 to 1196 m${}^{2}$.

**Figure 13:**
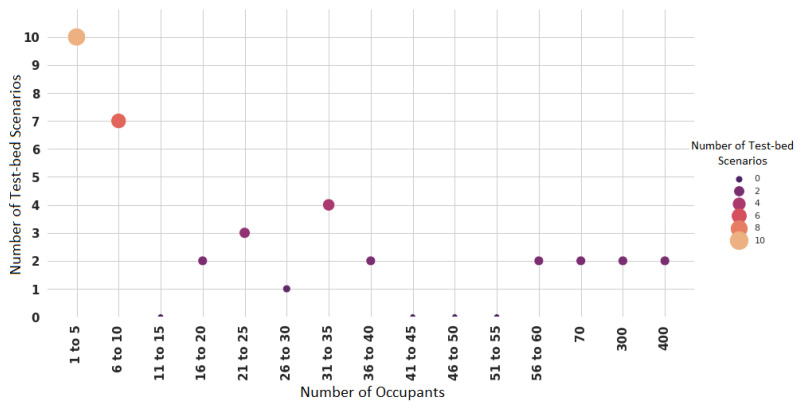
Test-bed scenarios by occupant capacity.

Other authors have described the enclosed spaces based on their occupancy capacity. Figure 13 presents the size of 21 places that were measured for their capacity. Offices and laboratories with a capacity of one to five occupants are the most used in the studies (10 test-bed places, representing 47.61%). These are followed by spaces with a capacity of six to 10 occupants (seven test-bed places, representing 33.33%), and laboratories with a capacity for 31 to 35 people (four test-bed places, representing 19.04%).

Regarding the number of sensors deployed in the enclosed space, there are researchers who have deployed around 240 sensors in an office and achieved 91% accuracy [65]. In contrast, there are other researchers who have installed a single sensor in an office with an area of 186 m${}^{2}$, obtaining 94% accuracy [51].

**Figure 14:**
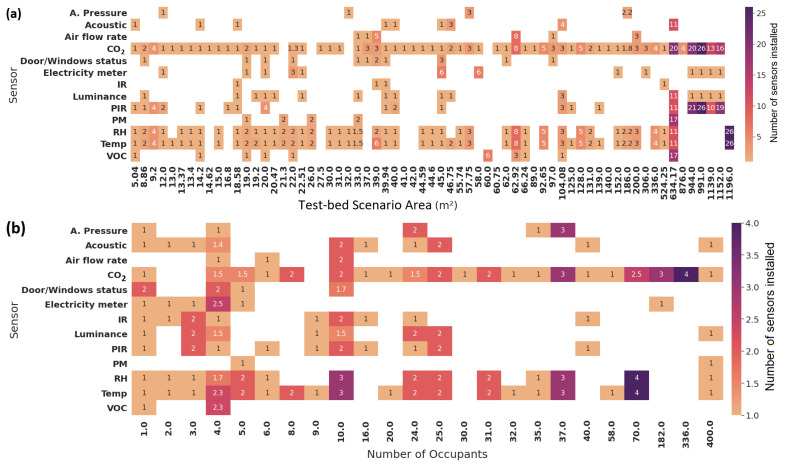
Type and total of sensors deployed. (**a**) Sensors installed by area. (**b**) Distribution of sensors installed by capacity.

Figure 14 depicts the number and type of sensors deployed per area (m${}^{2}$) and the number of occupants. Most of the studies (61.29%) are concentrated on collecting data from enclosed spaces with an area between 5 and 66 m${}^{2}$. The number of sensors and their type also varies depending on the author.

For instance, Han et al. [66] deployed a total of eight CO${}_{2}$, temperature, RH, and PIR sensors, as well as three VOC sensors in an office of 62.93 m${}^{2}$. In contrast, Szczurek et al. [67] installed only one sensor for CO${}_{2}$, temperature, and RH in a classroom with an area of 66.24 m${}^{2}$.

On the other hand, 24 studies (25.80%) have conducted experiments in places with areas between 300 and 990 m${}^{2}$, in which the number of installed CO${}_{2}$, temperature, RH, VOCs, PMs, PIR, acoustic, and light/luminescence sensors have increased. For example, Hobson et al. [68] installed a total of 26 CO${}_{2}$ and PIR sensors as well as one light/luminescence sensor and plug meter in a 991 m${}^{2}$ floor. Additionally, only three studies (3.22%) have considered large spaces (with an area of more than 1000 m${}^{2}$), in which the sensors used focused mainly on measuring CO${}_{2}$, temperature, and RH. These studies also had secondary sensors such as PIR, light/luminescence, and plug meters installed in smaller amounts.

### 3.4. Sensor Deployment Specifications

Sensor deployment, a method of placing sensors in the desired area, is considered a challenging issue for researchers and developers [69]. In wireless sensor networks (WSNs), sensor deployment is a fundamental problem to be solved as sensor deployment determines the coverage and connectivity of a WSN and its robustness against attacks. In addition, efficient sensor deployment can prolong the lifecycle of WSNs by reducing energy consumption [69].

**Figure 15:**
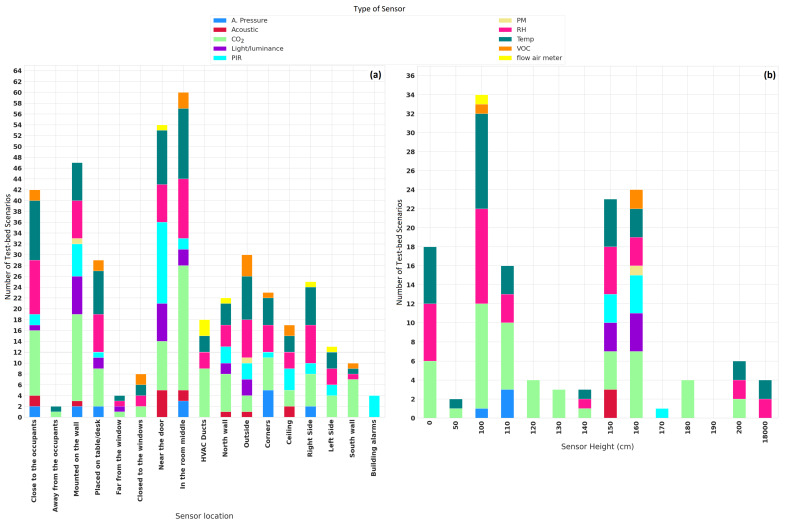
Specifications of the sensor deployment. (**a**) Placement of the sensors. (**b**) Installation height (cm) of the sensors.

Figure 15 illustrates the different installation locations and their heights. These have been extracted from 64 publications that share the details of the sensor deployment. The analysis is performed from enclosed spaces (92 places) in which the authors carried out their research.

It can be observed that the center of a room is the most common place to place CO${}_{2}$ sensors (23 scenarios, representing 25%), temperature sensors (13 scenarios, representing 14.13%), and RH sensors (11 scenarios, representing 11.95%). Furthermore, these sensors were installed close to the occupants of the place.

The CO${}_{2}$ sensor is mostly installed in HVAC ducts (nine scenarios, representing 9.78%), and PIR sensors are commonly installed near a door (15 scenarios, representing 16.30%). An easier way to place a sensor is by mounting it on a wall (17 scenarios, representing 18.47%) than placing it on a table (nine scenarios, representing 9.78%). It was also reported that few sensors were placed on the ceiling (three scenarios, representing 3.26%).

Regarding the height, CO${}_{2}$ (11 scenarios, representing 11.95%), temperature (10 scenarios, representing 10.86%), and RH (10 scenarios, representing 10.86%) sensors are usually placed at about 100 cm from the ground. CO${}_{2}$ sensors were also reported to have been placed at 110 cm and 160 cm (seven scenarios of each one, representing 7.60%, respectively) from the ground. In contrast, for the temperature and RH sensors (five scenarios of each one, representing 5.43%, respectively), the second most common height to place them is 150 cm from the ground.

### 3.5. Data Fusion

Several definitions of the term “data fusion” are presented in the literature. These definitions differ mainly based on the degree of generality and the specific research areas for which they have been used. One of the earliest and most popular definitions, at least in the multisensory area, was introduced by the Joint Directors of Laboratory and the US Department of Defense. According to them, data fusion is defined as: “A process dealing with the association, correlation, and the combination of data and information from single and multiple sources to achieve refined position and identity estimates, and complete and timely assessments of situations and threats as well as their significance” [70].

**Figure 16:**
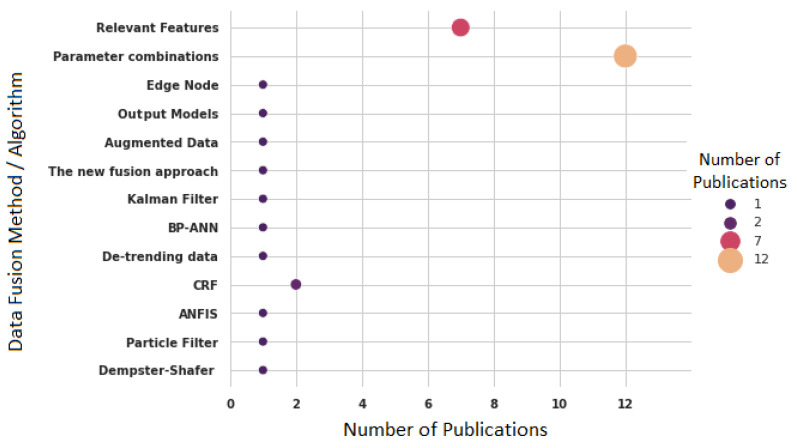
Data fusion methods/algorithms reported in the selected publications.

Figure 16 presents the methods and algorithms implemented in 26 studies that have specified the use of data fusion. Figure 16 unveils that parameter combinations (12 publications) are the most common used for sensor data fusion. The authors have combined different parameters to find the combination that achieves the best accuracy [4,57,71,72]. The second most used approach is the combination of relevant features (seven publications) obtained from Information Gain or Information Theory [41,73,74,75]. Chen et al. [76] have proposed to merge the output of data-driven models with occupancy models using a Particle Filter algorithm.

Alternatively, Das et al. [26] have developed a framework to fuse data at the edge node. The data are temporarily stored in a data stream buffer. Each piece of data retains its spatial–temporal properties in the buffer. Then, the fusion module correlates measurements of an entity from multiple points and reduces data redundancy. It uses a Kalman filter.

The most widely used algorithm is the conditional random field (CRF) (two publications), which is a relatively new type of discriminative probabilistic graphical model for labeling sequence data. Each feature is a real value and is associated with a numerical weight [16,77].

Almost all of the authors assert the improvement of the accuracy obtained from the models by using data fusion [25,66,68,78] except for Wang et al. [1], who state, “The fused dataset does not necessarily improve model accuracy but shows a better robustness for occupancy prediction”.

### 3.6. Algorithms and Datasets

**Figure 17:**
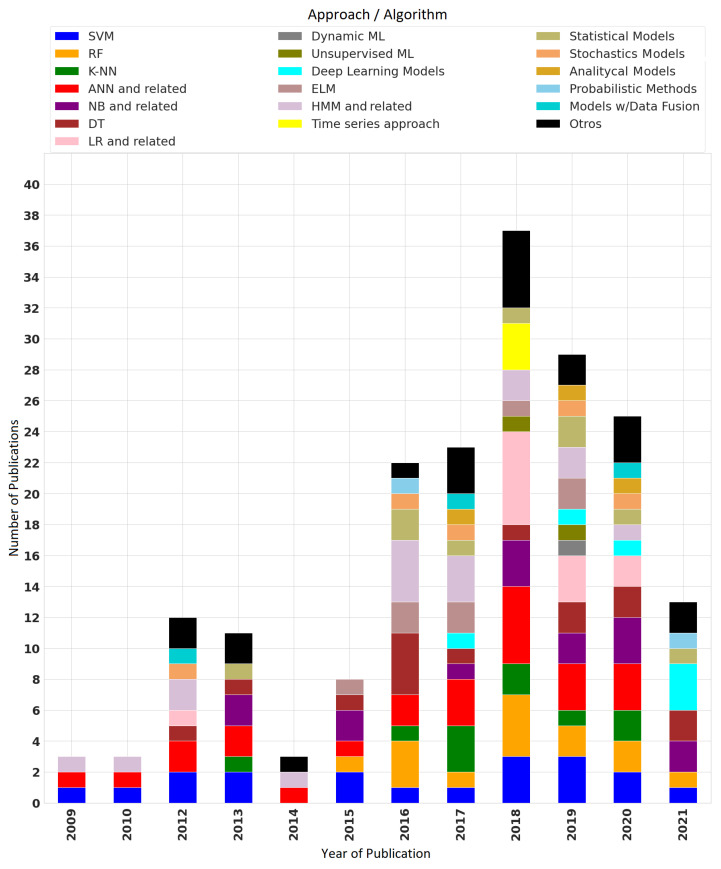
Occupational estimation approaches/algorithms over time.

Numerous occupational estimation approaches have been proposed and applied to various problems recently. Occupancy estimation provides information on the presence of occupants (whether or not they exist), occupancy density, the actual number of occupants, and individual occupant location [79]. The models extracted from the 93 selected publications include statistical, analytical, probabilistic, stochastic, and machine learning models. Figure 17 presents the trends in the use of models over the years per study.

SVM, which belongs to the Machine Learning approaches, has had a constant presence over the years (19 publications) and a greater application in 2018 and 2019. In the same way, the famous HMM statistical approach (11 publications) and the ANN have been frequently implemented in 24 studies, including their sub-models as well. For instance, Multi-Layer Perceptron (MPL), Feed-Forward Neural Network (FFNN), Neural Network with Random Weight (NNRW), Radial Basis Function Neural Network (RBFNN), Random Neural Network (RNN), Single-Layer FFNN (SLFFNN), Dynamic Time Delayed Neural Network Model (TDNN), and Artificial Neural Network with Bayesian Regulation Method (ANN-BRM) have had greater applications between 2017 and 2020.

Moreover, since 2015, interest in the Random Forest (RF) algorithm has increased, having 14 studies based on it. In contrast, only in 2018 and 2019, unsupervised ML algorithms and dynamic ML algorithms have emerged, showing that only a limited group of researchers has focused on them.

On the other hand, the datasets used to train each of the models have particular characteristics. The most important for this systematic review are the availability of data, whether the data have labels, and the time-stamp resolution in these works (see Table A1). Only 11 studies (11.82%) mention the availability of their datasets; that is, the data can be downloaded for experimentation by anyone. Regarding the labeled datasets, 87 studies (93.54%) have used data with labels to train and test their models. Only one study (1.07%) has used both labeled data and unlabeled data to carry out their experiments [13]. The developed models involve Linear Regression (LR), Instance-Bases learning with parameter k (IBK), RF, K-means, Hierarchical Cluster Analysis (HCA), Fuzzy C-means, and k-medoids, which provide an accuracy between 88.7% and 97.1%.

Of the five studies (5.37%) that used unlabeled datasets, three studies have developed models based on HMM, achieving accuracies of 90.24% [80] and between 89% and 91% [81,82] as well as Bayesian Networks (BN) [65]. On the other hand, a study developed unsupervised ML algorithms such as HCA and a logical flow chart [78], obtaining an error between 7% and 23%.

Finally, the most common time-stamp resolution is 1 min (25 publications, representing 26.88%), followed by 5 min (19 publications, representing 20.43%), and 15 min (12 publications, representing 12.90%).

## 4. Discussion

To answer RQ1, it is necessary to discuss not only the number of sensors but also the places in where they are installed. Each place has its own characteristics, not only in terms of its size but also in terms of the equipment present in it and its construction. This may be why researchers differ in choosing the number of sensors to be installed in spaces of similar size. For instance, in an office with an area between 19 and 22.5 m${}^{2}$, Diaz et al. [83] deployed two CO${}_{2}$ sensors, two temperature sensors, two RH sensors, one windows/door status sensor, and three plug meters, among other sensors. They used the CO${}_{2}$ concentration and the electricity consumption of the computer as indicators of the occupancy level. In a similar space, Candanedo et al. [71] installed one CO${}_{2}$ sensor, one temperature sensor, one RH sensor, and one light sensor. Their developed models included Linear Discriminant Analysis (LDA), Classification And Regression Tree (CART), RF, and Gradient Boosting Machines (GBM). Furthermore, the combination of parameters performed by Candanedo et al. obtained an accuracy between 32.68% and 99.33%.

In contrast, there are studies in which the area is larger than 100 m${}^{2}$, and fewer sensors are deployed. For example, Rastogi et al. [6] installed one CO${}_{2}$, temperature, RH, and infrared proximity sensor in a 524.5 m${}^{2}$ classroom. Their models included Multiple Linear Regression (MLR) and Quantile Linear Regression, and the coefficient of determination ($R^{2}$) for each model was 0.88 and 0.91, respectively. Jiang et al. [51] used a single CO${}_{2}$ sensor in a 186 m${}^{2}$ office, and they used the Feature Scaled Extreme Learning Machine (FS-ELM) model, which achieved 94% accuracy with a tolerance of four occupants difference.

According to the 93 selected publications (see Table A2), the authors mostly prefer to carry out their experiments in offices with an area between 5.03 and 62.92 m${}^{2}$, and between 97 and 634.17 m${}^{2}$, and in classrooms with an areas between 41 and 524.25 m${}^{2}$. These scenarios are easier to monitor because they are within the universities with which the researchers are affiliated. Fewer investigations have selected public spaces such as museums (1196 m${}^{2}$) [20,54], hospital rooms (33 m${}^{2}$) [57], cinema theaters (300 occupants) [46,47], and an elderly caring institution [59].

By analyzing the dimensions of the places, it is possible to make a size classification to define how many meters are a large or small space. To avoid the subjectivity of each person on the dimensions of the place, it makes sense to propose that spaces with an area between 1 and 70 m${}^{2}$ are small, whereas spaces with an area between 71 and 300 m${}^{2}$ are considered medium size. Finally, spaces with an area greater than 301 m${}^{2}$ should be considered large spaces.

Furthermore, using all the information extracted from the publications selected in this systematic review, it is possible to have an idea of the number of sensors to be installed using the proposed linear regression model presented in Equation (1), where *X* would be the value of the area in m${}^{2}$.

**Figure 18:**
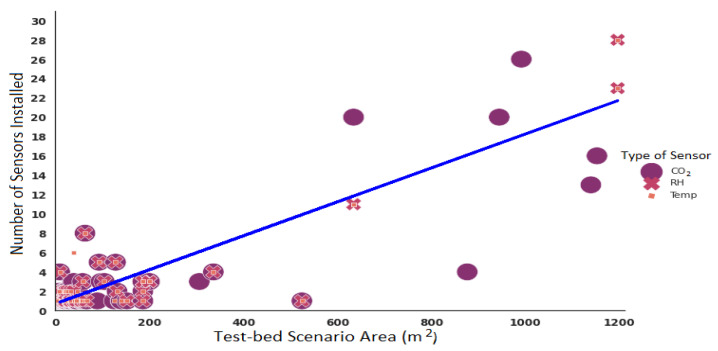
Proposed linear regression to calculate the number of sensor per area.

(1) y=0.0175X+0.7132,

To develop this linear regression, data were extracted from 66 publications that share the test-bed dimension in m${}^{2}$ and the number of sensors deployed in their experiments. Furthermore, it is important to point out that the sensors considered in these studies are only for measuring CO${}_{2}$, temperature, and RH. Figure 18 shows the proposed linear regression that obtained an $R^{2}$ of 0.757.

For example, the last column of Table A2 shows the results of the theoretical estimation of the number of sensors to be deployed using the proposed Equation (1). The results coincide with 30 studies included in this systematic review. In 12 publications, the estimation has a difference of one sensor versus the actual sensors deployed. Moreover, for investigations where space is large, the estimation is extremely close to the actual number of installed sensors. In other words, it is possible to figure out how many sensors to place according to the size of the selected space. Nevertheless, this equation does not ensure optimal sensing and will need to be tested with more scenarios to obtain reliable results.

To answer RQ2, the analysis shows that it is possible to obtain high accuracies using only environmental variables. In total, 38.70% of the publications use only environmental measures. For instance, Kampezidou et al. [84] have used one CO${}_{2}$ and temperature sensor in a 12.96 m${}^{2}$ room. Their study proposes an approach that includes a physics-informed pattern-recognition machine (PI-PRM) to detect occupancy, which achieves 97% accuracy. Vela et al. [12] carried out an indoor occupancy-level estimation by deploying one temperature, RH, and atmospheric pressure sensor in a university gym (33 occupants) and in a living room (32 m${}^{2}$). Their models involve SVM, k-NN, and DT, which obtained an accuracy between 95% and 97%.

The possibility of adding another type of sensor depends on the requirements, cost, and expected outcomes of the research. As for the optional sensors, the most widely used is the PIR, followed by the light and acoustic sensors.

On the other hand, answering the RQ3, the placement of sensors in an enclosed area can influence reliable data collection. Based on the selected studies, the most common locations for placement of CO${}_{2}$, temperature, and RH sensors are in the center of the room, ensuring that they are close to the occupants. Another option is to mount them on a wall or place them on a table. Moreover, the sensors are commonly installed 1 m from the ground.

Regarding RQ4, data fusion improves the models for occupancy detection or estimation. Most of the publications (83.87%) have used more than one type of sensor in their experiments. However, only 27.9% have explicitly specified the use of data fusion. The most used method is to combine parameters until an optimal combination is reached, which provides the highest accuracy. In addition, there are authors who have implemented more sophisticated methods, such as edge node fusion using Kalman Filter [26], Particle Filter [76], ANFIS [27], and BP-ANN [85]. All publications have shown that data fusion improves the accuracy of models to detect or estimate the occupancy, except for one study, which contradicts the benefits of data fusion [1].

Finally, to answer RQ5, it is important to discuss the models as well as the datasets used to train them. From the extracted information, it was discovered that Supervised Machine Learning Algorithms such as SVM, RF, DT, and ANN are very popular among researchers in addition to the models based on the Bayes Theorem and HMM. Since 2016, very few authors have carried out experiments using HMM and unlabeled data to estimate/detect indoor occupancy. In contrast, unsupervised and Dynamic Machine Learning models are of little interest to researchers so far. There is not even a single study where Semi-Supervised Machine Learning models have been used.

For instance, Crivello et al. [13] presented a system that is able to perform room occupancy detection and social interaction identification, using data coming from both energy consumption information and the environment (temperature and RH). Their aim was to determine room occupancy status and to detect socialization events in the monitored room. In order to use unsupervised methods, their approach relied on a minimal set of domain-based knowledge, such as the number of workers assigned to each room and the fact that, during each day, most of the time spent by them is on performing a usual daily activity which involves social interactions. The unsupervised clustering techniques implemented were K-medoids, K-means, hierarchical clustering, and fuzzy C-means. These four methods have a fixed number of clusters: two clusters when the goal is room occupancy detection and three clusters when the interest is in the identification of social interactions. The accuracy obtained for occupancy detection in their study was between 88.7% and 97.1%, and for socialization, it was between 93% and 95.4%.

With these investigations, it is clear that it is possible to use data without labels to detect/estimate occupancy in enclosed spaces. Only five authors have ventured into this field, which allows for reducing costs and time in data collection.

## 5. Conclusions

This systematic review presented a discussion on occupancy estimation/detection, sensor deployment, and a possible way to set the number of sensors, depending on the area of the enclosed space. The aim is to help researchers and practitioners to identify the most viable sensor placement to detect and estimate occupancy according to their objectives and performance demands.

After the implementation of the inclusion and exclusion criteria to the articles discovered in the SCOPUS database, 93 articles from 2009 to 2021 were considered and discussed. The selected studies allowed achieving the objectives and answering the research questions of this systematic review. Most of the studies (21.5%) were conducted in the USA. Other contributions were from Singapore (10.75%) and China (9.67%). After analysis of the described keywords, it was discovered that the keyword “carbon dioxide” has the highest frequency of 43. Other keywords with a high frequency include “occupancy detection” (24), “learning system” (21), “machine learning” (21), and “energy efficiency” (20). A summary of the findings of this systematic review is presented according to each research question: RQ1: Most of the studies (61.29%) are concentrated on collecting data from offices with an area between 5 and 66 m${}^{2}$. However, the number of sensors used in these studies depends on the author. Therefore, a linear regression model is proposed as a tool to calculate the number of sensors to be deployed according to the dimensions of the place.

RQ2: The results show that 90.32% of the total studies considered include CO${}_{2}$ sensing as the main environmental parameter. However, 4.30% of the studies consider temperature and RH as priority measures.

RQ3: In total, 68.81% of the publications share the details of the sensor deployment from 92 places where the authors have conducted their research. The researchers preferred placing sensors that measure CO${}_{2}$, temperature, and RH in the middle of the room, at a height of 100 cm from the floor. Furthermore, it is sought that the installation of these sensors is close to the occupants.

RQ4: Regarding data fusion, only 27.95% of the studies specified the use of data fusion methods and unveiled that parameter combination is the most used method, which is followed by the combination of relevant features.

RQ5: In total, 20% of research works preferred Machine Learning algorithms such as SVM (20.43%), followed by RF (15.05%) and ANN (12.90%), including their sub-models as well. Results show that five publications specify the use of unlabeled data to detect/estimate indoor occupancy. However, the implementation of unsupervised models using environmental variables is almost unexplored.

Future research should focus on exploring models that can use unlabeled or semi-labeled data in order to conduct further research on these approaches. Furthermore, it is important to study other methods to fuse data. The current studies have made use of the most basic level of data fusion. Finally, the development of a tool to set the number of sensors to be installed is important to do as well as the evaluation of the linear regression proposed in this systematic review. This would allow a cheaper but trustworthy development of experiments.

Even though all the answers were obtained, the current study also has limitations. The defined inclusion and exclusion criteria limit the scope of this study. Consequently, this systematic review does not provide details about monitoring systems that do not involve environmental parameters. Furthermore, the publications were obtained from only one database (SCOPUS), and the applied search restriction was for publications from 2009 to September 2021.

## Figures and Tables

**Table 1:** Inclusion and exclusion criteria.

	Inclusion Criteria		Exclusion Criteria
IC1	Publications whose titles contain the word “occupancy” and at least one environmental variable (e.g., CO${}_{2}$, Temperature, RH, etc.) considered.	EC1	No match to the inclusion criteria.
IC2	Articles that contain keywords that match the defined keywords.	EC2	Duplicate publication.
IC3	The abstracts include search keywords or have a detectable relationship with the selected theme.	EC3	Research that involves datasets from other authors.
IC4	Articles that include at least one environmental sensor in their experiments.	EC4	Thesis, books, and preprint studies.
IC5	The publication is available in full text in an open manner or through any of Tecnologico de Monterrey’s subscriptions.		

**Table 2:** Summary of selected studies.

Query No.	Query Strings	Results	Selected
1	KEY ((“occupancy” OR “occupancy estimation” OR “occupancy detection” OR “occupancy building” OR “occupancy levels”) AND ((“Ambient” AND (“sensing” OR “Variables”)) OR (“environmental” AND (“sensor” OR “variables” OR “parameters”))))	153	33
2	TITLE-ABS-KEY (((indoor OR enclosed) AND (occupancy) AND ((environmental OR environment) AND (sensor OR variables OR parameters))))	623	15
3	ALL ((indoor) OR (enclosed)) AND ((occupancy AND (estimation OR detection OR prediction))) AND ((environmental AND variables) OR (environmental AND sensing) OR (non-intrusive))	3031	45
	Total	3807	93

## Data Availability

Not applicable.

## Abbreviations

The following abbreviations are used in this manuscript:

A.F.R/A.V	Air Flow Rate/Air Volume
ADTree	Alternating Decision Tree
ANFIS	Adaptive Neuro-Fuzzy Inference System
ANN	Artificial Neural Network
ANNBRM	Artificial Neural Network with the Bayesian Regulation Method
ARHMM	Autoregressive Hidden Markov Model
ANN-PCA	Artificial Neural Network with one Principal Component Analysis step
BE	Bayesian Estimation
BMCMC	Bayesian Markov Chain Monte Carlo
BN	Bayesian Network
BR	Bayesian Ridge
BTM	Bagged Tree Model
CA	Correlation Analysis
CAM	Count Prediction Based on Data from 3D Stereovision Camera
CART	Classification And Regression Tree
CDBLSTM	Convolutional Deep Bi-Directional Long Short-Term Memory
CRF	Linear chain Conditional Random Field
CS	Cosine Similarity
Decision Stump	Decision Stump
DFE	Flow Detection Engine
DT	Decision Tree
D/W	Door/Windows status sensor
Dynamic ML	Dynamics Machine Learning strategies
ELM	Extreme learning machine
E.M	Electricity plug meter
EV	Ensemble voting
EWMA	Exponentially Weighted Moving Average
FFNN	Feed-Forward Neural Network
Flow Chart	Flow Chart
FML	Frequentist Maximum-Likelihood
FS-ELM	Feature scaled Extreme Learning Machine
Fuzzy C-means	Fuzzy C-means
GAFK	Genetic Algorithm—unscented Kalman Filter
GBM	Gradient Boosting Machines
GB	Gray-Box model
GP	Gaussian Processes
HAC	Hierarchical Cluster Analysis
HMM	Hidden Markov Model
HMSM	Hidden Markov-Switching Model
IBK	Instance-Bases learning with parameter k
IHMM	Inhomogeneous Hidden Markov Model
IHMM-MLR	Inhomogeneous Hidden Markov Model with Multinomial Logistic Regression
J48	A decision tree classification algorithm based on Iterative Dichotomizer 3
LAHMM	Location-Aware Hidden Markov Model
LBE	Learning-by-Examples
LDA	Linear Discriminant Analysis
LR	Linear Regression
LRF	Local Receptive Fields
LSTM	Long Short-Term Memory
LTP	Law of Total Probability
MAP-HMM	Maximum a Posteriori Probability estimation of Hidden Markov Model
MBM	Mass Balanced Models
MLR	Multiple Linear Regression model
MLP	Multi-Layer Perceptron
MSPRT	Multiple-Hypothesis Sequential Probability Ratio Test
NB	Naïve Bayes
NFA	New Fussion Aproach
NMF-ELSR	Non-Negative Matrix Factorization with Ensemble Least Square Regression
NNRW	Neural Network with Random Weight
NP strategy	Non-Parametric strategy
P strategy	Parametric strategy
PEA	Point Extraction Algorithm
PEM	Prediction Error Minimization
PI-PRM	Physics-Informed Pattern-Recognition Machine
PnP	The novel Plug-and-Play Method
QDA	Quadratic Discriminant Analysis
QL	Quantile Regression
RBE	Rule Based Engines
RBF-NN	Radial Basis Function Neural Network
RBH	Rule-Based Heuristic
REPTree	A fast decision tree learner
RF	Random Forest
RH	Relative Humidity
RIG-ELM	Rig Extreme Learning Machine
RNN	Random Neural Network
RUP-STD	Room Utilization Prediction with carbon dioxide sensor—Seasonal
	Trend Decomposition
RUP-STL	Room Utilization Prediction with carbon dioxide sensor—Seasonal
	Trend decomposition based on Loess
SD-HOC	Seasonal Decomposition for Human Occupancy Counting
SDE	Stochastic Differential Equations
SDLM	Sequential Deep Learning Model
SGF	Savitzky–Golay Filter
SLFFNN	Single-Layer Feed-Forward Neural Network
SMO	Sequential Minimal Optimization
SSA	Steady-State Approx
SURE	Stein’s Unbiased Risk Estimator
SVM	Support Vector Machine
TAN	Tree Augmented Naïve Bayes
TDNN	Dynamic Time Delayed Neural Network Model
TM	Transient Method
TSE	Triangular Shape Extraction
WRANK-ELM	Wrank Extreme Learning Machine
Yolo v4	Yolo v4 tiny model
ZeroR	Zero Rule

## Appendix A

**Table A1:** Datasets characteristics and algorithms developed.

Study	Sensed Time	Occ. Resol.	Data Avail.	Labels	Time-Stap Resol.	Algorithm	Results
[25]	2 Y	Detection	NO	YES	1 min	LTP, NB, CART	Accuracy 90.9–93.5%
[86]	10 D	Num. People	NO	YES	15 min	Yolo v4, BTM	Accuracy 99.5%
[87]	15 D	Detection	NO	YES	1 min	LSTM	Accuracy 96.8%
[84]	21 D	Detection	NO	YES	5 seg	SGF, SURE, PI-PRM	Accuracy 97%
[88]	60 D	Detection	NO	YES	5 min	SDLM, LSTM, RF, SVM	Accuracy 63–70%
[18]	20 D	Num. People	NO	YES	1 min	BMCMC	Accuracy 43.5%
[89]	20 D	Num. People	NO	YES	15 min	K-NN, GP, RF, BR, MLP	MAE 0.21–1.84
[26]		Detection, Levels	NO	YES	15 sec	SVM, LR, CS	Accuracy 96.9–97.95%
[90]	120 D	Num. People	YES	YES		CA	
[66]	6 D	Detection, Num. People	NO	YES	1 min	NFA	Accuracy 98.3%
[7]	30 D	Detection, Levels	NO	YES	1 min	Bayes filter	Accuracy 85.57%
[2]	11 H	Num.People	NO	YES	1 seg	DFE	Recall 91.93–96.80%
[6]		Num.People	NO	YES	1 min	MLR, QL	$R^{2}$ 88–91%
[12]	13 D	Levels	YES	YES	10 seg, 30 seg, 1 min	K-NN, SVM, DT	Accuracy 88.39–99.67%
[48]	30 D	Num. People	NO	YES	1 min	GcForest	Accuracy 86%
[11]	4 D	Detection, Levels	NO	YES	5 min	FNN	Accuracy 83.6–94.3%
[65]	10 D	Detection, Num. People	YES	NO	30 min	BN	Accuracy 82–91%
[91]	15 D	Num.People	NO	YES	1 min	RF, ELM	RMSE of RF 2.75–10.44 Accuracy of ELM 67.92–69.17%
[63]	1 D	Detection	NO	YES		Dynamic ML	
[68]	12 D	Num. People	NO	YES	5 min	ANN, MLR	$R^{2}$ 96.5–97.5%
[78]	45 D	Levels	NO	NO	1 min	HCA, logical flow chart	Error 7–23%
[19]	15 D	Detection	NO	YES	10 seg	SVM	Precision 87%
[85]	14 D	Num. People, Levels	NO	YES	5 min	ANN, MLR	MAE 2.15–3.40, F1-score 73.57–84.36%
[3]	120 D	Num. People	NO	YES	10 min	FML, BE, ELM	NRMSE 0.2230–0.2470
[10]	4 D	Num. People	NO	YES	5 min	SDE	Accuracy 88–94%
[22]	37 D	Detection	YES	YES	5 min	LR, LDA, K-NN, CART, NB, SVC, RF, GB	Accuracy 79–85%
[92]	15 D	Detection, Num. People	YES	YES	10 seg	LR, SVM, ANN	F-score 24.43–25.15%
[15]		Num. People	NO	YES	5 seg	SMO, HMM, IBK, RF, J48, Bagging, REPTree, NB, Decision Stump	Accuracy 8.66–90.1%
[93]	90 D	Num. People	NO	YES	30 seh	LR	Accuracy 90–95%
[94]	7 D	Levels	NO	YES	1 min	CA	
[13]	90 D	Detection, socialization	NO	YES/NO	5 min	LR, IBK, RF, K-means, Hierarchical, Fuzzy C-means, k-medoids	Accuracy 88.7–97.1%
[14]	4 D	Detection, status (alive or dead)	NO	YES	1 min	PEA, TSE	
[52]	7–10 h	Num. People	YES	YES	30 min	Proxy model	RMSE 60.44%
[62]	12 D	Detection, status (active or not)	NO	YES	30 min	MLR	Accuracy 50–99.8%
[58]		Num. People	NO	YES		ELM	RMSE 4.83–6.64
[1]	9 D	Num. People	NO	YES	5 min	K-NN, ANN, SVM	MAE 2.3–2.6
[75]	49 D	Detection, Num. People	YES	YES	15 seg	ZeroR, JRip, NB, J48, LR, K-NN, RF	Occ. Accuracy 50.8–75.1%, Num. People. Accuracy 42.7–64.3%
[83]	7 Y	Detection, Levels	NO	YES	1 min	CO${}_{2}$ concentration and computer electricity consumption indicators of occupancy leves	Error 0.25–73.71%
[77]	1 Y	Detection, Num. People	NO	YES	5 seg	CRF	Detection Accuracy 84–98%, Num. People. NRMSE 0.105–0.15
[24]	4 D	Num. People	NO	YES	15 min	RNN	Accuracy 88%
[8]	31 D	Levels	NO	YES	15 min	WRANK-ELM, RIG-ELM	Accuracy 75.63–79.17%
[95]	8 D	Detection	NO	YES	4 min, 20 min	PnP	MAE 0.002–0.54
[96]	30 D	Num. People	NO	YES		GAKF, CAM	NRME 0.075–0.71
[67]	16 D	Num. People	NO	YES	3 min	K-NN, LDA	MCR 1.58–3.27%
[9]	30 D	Levels	YES	YES	15 min	ELM-LRF	Accuracy 77.27%
[45]	120 D	Num. People	NO	YES	5 min	MBM, ANN, PEM, SVM	RMSE 12.1–27.4
[71]	1 D	Detection	YES	YES		LDA, CART, RF, GBM	Training Accuracy 83.38–100% Testing Accuracy 32.68–99.33%
[72]	90 D	Levels	NO	yes	5 min	RF	Accuracy 71–95.9%
[51]	30 D	Num. People	NO	YES	1 min	FS-ELM	Accuracy 94%
[16]	90 D	Num. People, Detection, Levels	NO	YES	10 min	CRF, HMM	Acuracy 85–93%
[74]	16 D	Levels	NO	YES	30 min	C4.5	F1-Score 0.47–0.65
[57]	210 D	Num. People	NO	YES	5 min	The Beam-break method, and the CO${}_{2}$ method	test *p* > 0.05
[17]	2 D	Levels	NO	YES	20 seg 5 seg	HMM, ARHMM	Accuracy 25.2–84%
[54]	1 Y	Detection, Levels	NO	YES	10 seg	RBE, LBE	Accuracy 82%
[56]	7.8 H	Detection	NO	YES	10 min	BE, CA	Accuracy 66%
[4]	20 D	Detection, Num. People	NO	YES	1 min	SVM, K-NN, ANN, NB, TAN, DT	Local occ. RMSE 0.109–0.311 Global occ. RMSE 0.211–1.192
[27]		Levels	NO	YES	2 min, 10 min	ANFIS	
[97]	90 D	Num. People	NO	YES	20 seg	ARHMM, SVM, HMM	RMSE 0.94–1.08
[98]	20 D	Num. People	NO	YES	1 min	RBF-NN	Accuracy 86.50–88.74%
[73]	300 D	Num. People	NO	YES	1 min	SVM, ANN, HMM	Accuracy 65–75%
[41]	300 D	Num. People	NO	yesYES	20 min	SVM, ANN, HMM	Accuracy 70–75%
[53]		Detection	NO	YES	15 min	EWMA	Accuracy 83.33–87.03%
[46]	1 M	Num. People	NO	YES	5 min	SD-HOC	Accuracy 93.71–97.73%
[47]	1 M	Num. People	NO	YES	5 min	RUP-STD, RUP-STL, SVM, NMF-ELSR	Accuracy 69.96–99.52%
[99]	31 D	Levels	NO	YES	15 min	CDBLSTM	Accuracy 76.04%
[80]	43 D	Num. People	YES	NO	5, 10, 20, 30 min	HMM	Accuracy 90.24%
[76]	32 D	Levels	NO	YES	15 min	ELM, SVM, ANN, K-NN, LDA, CART	Accuracy 81.25–93.45%
[21]	8 M	Levels	NO	YES	1 min	SVM, NB, TAN, ANN, RF	Error 9.2–18.2%
[100]	1 M	Levels	NO	YES	5 min	ELM	Accuracy 81.37%
[20]		Levels	NO	YES	10 seg	SVM	Occ. Index approx. 51%
[101]	10 W	Levels	NO	YES	1 min	ADTree	The correlation 48.05% for acoustic, 35.70% for CO${}_{2}$, 32.49% for RH, and 11.98% for temperature.
[102]		Detection, Num. People	NO	YES	15 min	RBH, MLP, GP, LR, SVM, EV	Accuracy 46–92%
[99]	31 D	Detection	NO	YES	15 min	IHMM-MLR	Accuracy 78.13%
[23]	10 H	Levels	NO	YES	1 seg	NNRW	Accuracy 52–100%
[103]	33 H, 10.4 H, 4 H	Num. People	NO	YES	7.5 seg	SMO, HMM, IBK, RF, J48, Bagging, REPTree, NB, DecisionStump	Accuracy 46.6–99.9%
[104]	102 D	Num. People	NO	YES	15 min	SSA, TM, FFNN	NMSE 0.23–1.60
[105]	28 D	Num. People	NO	YES	1 min	IHMM, GcForest	EA% 74.3–83.3
[49]	9 W	Num. People	NO	YES	5 min	GB	RMSE 0.66–0.77
[106]	9 M	Num. People	NO	YES	15 min	CART, SMV	Accuracy 93.84–95.59%
[50]	1 M	Detection	NO	YES	5 min	HMSM	Accuracy 75.5–96.5%
[107]	7 D	Detection	NO	YES	1 min	SLFN	Accuracy 99.79%
[61]	56 D	arrival time—departure time—number of People	NO	YES		ANNBRM	$R^{2}$ 92%
[108]	7 D	Levels	NO	YES	3 min	LAHMM	Accuracy 90%
[59]	1 Y	Detection	NO	YES	10 min	Adaboost, C5.0, SVM, QDA, ANN-PCA	Accuracy 80%
[109]	34 D	Levels	NO	YES	30 min	C4.5, RF	Accuracy 86–88%
[60]	7 D	Num. People	NO	YES	1 min	CART, HMM	F-statistic 24
[82]	1 M	Detection	NO	NO	5 min	HMM	
[110]	6 M	Num. People	NO	YES	1 min	TDNN	RMS 0.684–0.811
[111]	1 M	Levels	YES	YES	5 min	P-strategy, NP-strategies, SVM, ANN	Accuracy 81.1–88%
[112]	2 W	Levels	NO	YES	1 min	MAP-HMM, MSPRT, ANN	RMSE 1.2–2
[81]	10 D	Levels	NO	NO	30 min	HMM, BN	Accuracy 89–91%
[55]	56 D	Detection, Levels	NO	YES	10 seg	MLP, K-NN, DT, RF	F1 scores 0.15–0.94
[113]	18 D	Detection	NO	YES	1 min	k-NN	Accuracy 74.51–97.36%

**Table A2:** Resume of the sensors deployed, place, place dimension, and theoretical estimation of the sensors to deploy.

Study	Sensors														Place Type	Place Size		Est. Sensors to Deploy
	CO${}_{2}$	Tem	RH	A. Press	VOCs	PMs	A.F.R/A.V	PIR	IR	Acous.	Light/Lum.	D/W	Elec. Meter	Other		m${}^{2}$	Num. Occ	
[25]	2	2	2		1							1	1	1	Office	19	5	1
[86]	1	1	1		1						1	1		1	Kitchen Apartment	20		1
[87]	1	1	1					1			1				Office			0
[84]	1	1													Room	12.96		1
[88]	1	1	1												Office	31		1
	1	1	1												Office	30		1
	1	1	1												Office	15		1
[18]	3						1					1			Office	37		1
	3						1					1			Office	97		2
[89]	2	2													Office		8	0
[26]	4	4	4					4							Room	9.2		1
[90]	4	4	4												Large Classroom	336		6
	2	2	2												Medium Classroom	131		3
[66]	8	8	8		3			8							Office	62.92		2
[7]	1														Office	186		4
[2]	1	1	1			1				1	1			5	Indoor Univ. Hallway		400	0
	1	1	1			1				1	1			5	Outdoor Food Court		400	0
[6]	1	1	1						1						Classroom	524.25		10
[12]		1	1	1											University Gym		35	0
		1	1	1											Living Room	32		1
[48]	1														Office	14.62		1
[11]	1	1	1	1	1			1		1	1	2		0	Secretary’s Section		1	0
	1	1	1	1	1			1		1	1	2		0	Office		4	0
[65]	30	30	30					30		30	30	30	30		Office			0
	5	5	5					5		5	5	5	5		Apartment			0
	1	1	1					1		1	1	1	1		House			0
[91]	1													1	Classroom		70	0
	1													1	Clasroom		40	0
	1													1	Study Zone		30	0
	1													1	Study Zone		35	0
[63]	1	1	1					2					1		EEBLab	12		1
[68]	26							26			1		1	3	Floor A	991		18
	13							10			1		1	5	Floor B	1139		20
	20							21			1		1	2	Floor C	944		17
	16							19			1		1	4	Floor D	1152		21
[78]	2	2	2		1							1	1	1	Office	19		1
[19]	2	2	2					2		2	2				Classroom		25	0
[85]	3	3	3				3								Office	200		4
[3]	1	1	1										1		Office	152		3
[10]	1														Office	30		1
	1														Office	42		1
[22]		1	1												Office	12		1
[92]	2	2								3	1				Office	46.75		1
[15]	1	1	1											2	Office	13.37		1
	1	1	1											2	Office	44.59		1
	1	1	1											2	Office	55.74		1
[93]	2	2	2						2						Classroom			0
[94]	4	4	4												Lab		70	0
	2	2	2												Lab		31	0
[13]		1	1						1	1			1	1	Office		3	0
		1	1						1	1			1	1	Office		2	0
		1	1						1	1			1	1	Office		1	0
[14]		2	2			2									7th Floor House	26		1
		2	2			2									1st Floor House	21.3		1
		2	2			2									Office	33		1
[52]	1														Meeting Room	140		3
[62]	1	1	1					4				1	1	1	Control Testbed	20		1
[58]	1														Bus			0
[1]	3	3	3											3	Office	200		4
[75]	1	2	2		1										Apartment 1	22		1
	1	2	2		1										Apartment 2	22		1
	1	2	2		1										Apartment 3	22		1
	1	2	2		1										Apartment 4	22		1
[83]	2	2	2									1	3	3	Office	22		1
	2	2	2									1	3	3	Office	22		1
[77]	1							1	1	1	2			4	Kitchen		24	0
		1						1	1		1				Researcher’s Office		9	0
		1						2	3		2			2	Office		3	0
[24]	2	2	2					1			1	1			Chamber	8.86		1
	1	2	2					1			1	1			Office		10	0
[8]	3	3	3	3											Office	186		4
[95]	1	1	1		1			1		1					Office	5.04		1
	1	1	1		1			1		1					Living Room Apartment	14.2		1
[96]	1	1						1						4	Study Zone	125		3
	1	1						1						4	Classroom	139		3
[67]	1	1	1												Clasroom	66.24		2
[9]	2	2	2	2											Lab		24	0
[45]	4														Lecture Theatre	876		16
[71]	1	1	1								1				Office	20.47		1
[72]	1	1												2	Seminar Room CP103		20	0
	1	1												2	Classroom CP106		58	0
	1	1												2	Classroom CP108		58	0
[51]	1														Office	186		4
[16]	1							1	1	1					Meeting Room		16	0
	1							1	1	1				2	Kitchen		40	0
	1							1	2					4	Office		10	0
	1							1	1	1					Open Space		4	0
[74]	1	1	1					1		1	1	3	6		Office	45		1
[57]	1	1	1				1				1	1			Hospital Rooms	33		1
[17]	4	4	4				2	4				2		1	Lab		10	0
[54]		28	28												Museum	1196		21
[56]						8	8								Main Corridor			0
[4]	1	1	1					1	1	1	1	1		1	Office	18.58		1
	1	1	1					1	1	1	1	1		1	Office	39.94		1
[27]	3	3	3		1			3		4	3			4	Office	104.08		2
[97]	1	1	1				1	1						1	Lab		6	0
[98]	1	1	1					2		1	1				Lab 1	40		1
	1	1	1					2		1	1				Lab 2	40		1
[73]	20	11	11		17	17		11		11	11				Open Office	634.17		12
[41]	20	11	11		17	17		11		11	11				Open Office	634.17		12
[53]		1	1												Office	12	3	1
[46]	1														Academic Staff Room	12		1
														1	Cinema Theatre		300	0
[47]	1														Room	12		1
														1	Cinema Theatre		300	0
[99]	2	2	2	2											Lab	186		4
[80]	5	5	5												House First Floor	128		3
	5	5	5												House Second Floor	92.65		2
[76]	1	1	1	1											Lab	186		4
[21]	1	1	1					1		1	1	1			Office	0		0
[100]	3	3	3	3											Tutorial Room	57.75	37	1
[20]		23	23												Museum	1196		21
[101]	20	11	11		17			11		11	11				Open Office	634.17		12
[102]	2									2	2	2			Lab 1		4	0
	2									2	2	2			Lab 2		10	0
[99]	3	3	3	3											Lab	186		4
[23]	1														Classroom	60.75		1
[103]	1	1	1											5	Room 1	13.4		1
	1	1	1											5	Room 2	44.6		1
	1	1	1											5	Room 3	55.74		1
[104]	3												1		Univeristy Auditorium	306	182	6
[105]	1														Office	14.62	4	1
[49]	1														Office 1	40	6	1
	1														Office 2	27.5	5	1
[106]	1	1	1								1		1		Office	22.51		1
[50]	1														Summerhouse	89		2
	1														Classroom	41		1
[107]	1							1							Office	16.8		1
[61]	1	2	1								1				Office	19.2		1
[108]	1	5	5					5		5	1				Apartment			0
[59]	5	5	5												Elderly Caring Institution			0
[109]	2	3	2		4		1			1	2	3	4		Office		4	0
[60]	1	1	1					4			1				BICT	20		1
[82]	1	1	1										1	1	House	62		1
[110]	3	6	2				5		1			2			Office	39		1
[111]	1	1													Lab		32	0
[112]	1												6		Office	58		1
[81]	2	3	2		2			1		1	2	3	4		Office		4	0
[55]					6									2	KIT-ESHL	60		1
[113]	1	1	1												Clasroom	66.24		2

The column Num. Occ. refers to the place dimension based on number of persons capacity.
